# Kidney Organoid and Microphysiological Kidney Chip Models to Accelerate Drug Development and Reduce Animal Testing

**DOI:** 10.3389/fphar.2021.695920

**Published:** 2021-07-26

**Authors:** Wei-Yang Chen, Eric A Evangelista, Jade Yang, Edward J Kelly, Catherine K Yeung

**Affiliations:** ^1^ Department of Pharmacy, School of Pharmacy, University of Washington, Seattle, WA, United States; ^2^ Department of Pharmaceutics, University of Washington School of Pharmacy, Seattle, WA, United States; ^3^ Kidney Research Institute, University of Washington School of Medicine, Seattle, WA, United States

**Keywords:** kidney, microphysiologic system, organoids, translational sciences, microfluidic

## Abstract

Kidneys are critical for the elimination of many drugs and metabolites via the urine, filtering waste and maintaining proper fluid and electrolyte balance. Emerging technologies incorporating engineered three-dimensional (3D) *in vitro* cell culture models, such as organoids and microphysiological systems (MPS) culture platforms, have been developed to replicate nephron function, leading to enhanced efficacy, safety, and toxicity evaluation of new drugs and environmental exposures. Organoids are tiny, self-organized three-dimensional tissue cultures derived from stem cells that can include dozens of cell types to replicate the complexity of an organ. In contrast, MPS are highly controlled fluidic culture systems consisting of isolated cell type(s) that can be used to deconvolute mechanism and pathophysiology. Both systems, having their own unique benefits and disadvantages, have exciting applications in the field of kidney disease modeling and therapeutic discovery and toxicology. In this review, we discuss current uses of both hPSC-derived organoids and MPS as pre-clinical models for studying kidney diseases and drug induced nephrotoxicity. Examples such as the use of organoids to model autosomal dominant polycystic kidney disease, and the use of MPS to predict renal clearance and nephrotoxic concentrations of novel drugs are briefly discussed. Taken together, these novel platforms allow investigators to elaborate critical scientific questions. While much work needs to be done, utility of these 3D cell culture technologies has an optimistic outlook and the potential to accelerate drug development while reducing the use of animal testing.

## Introduction

The approval rate of new drugs that complete clinical trials and reach the market is just 14% despite promising preclinical safety and efficacy data obtained using 2-dimensional (2D) human cell culture and live animal studies. (Tagle, 2019; Wong et al., 2019; Sura et al., 2020). In addition to providing data that may not translate to humans, traditional methods often require the use of live animals, which is expensive and ethically complex. As such, the United States Environmental Protection Agency has prioritized reduction of mammalian studies by 2035 and the United States Food and Drug Administration has issued guidance to support the 3Rs (replace/reduce/refine) for animal testing. In response, emerging technologies incorporating engineered three-dimensional (3D) *in vitro* cell culture models, such as organoids and microphysiological systems (MPS) culture platforms have been developed to enhance efficacy, safety, and toxicity evaluation of investigational compounds.

Kidneys are responsible for the elimination of many drugs and metabolites via the urine. In addition, the kidneys play a critical role in filtering waste and maintaining proper fluid and electrolyte balance (Bajaj et al., 2018). Kidney organoids and kidney MPS have been developed to reproduce normal kidney functions; the primary goal is for these systems to replicate specific regions of the nephron, including, podocytes in glomerular capillaries, or epithelial cells in proximal tubules, loops of Henle, and distal convoluted tubules. Organoids are three-dimensional self-assembled constructs that contain multiple cell types which are structurally and functionally representative of their source tissue. While they have been used as research tools for more than 50 years, their utility has been primarily in studying developmental biology (Lancaster and Knoblich, 2014; Clevers, 2016). In recent years, advances in stem cell reprogramming have seen human pluripotent stem cell (hPSC)-derived organoids used in biomedical research alongside 2D cell cultures and animal models. Many of the benefits are derived from the ability to maintain biomimetic interactions among multiple cell types. Kidneys, for example, contain at least 26 interacting cell types (Al-Awqati and Oliver, 2002; Quaggin, 2016); such complexity is difficult to recapitulate in 2D cultures. Organoids provide a method to study cellular responses, biochemical processes, and responses in the presence of multi-cellular interactions. MPS are microfluidic devices capable of mimicking biological function of organs including liver, gut, lung, and brain and are receiving tremendous attention in drug development (Chang et al., 2017; Giordano et al., 2021). MPS have evolved into scalable and reproducible multi-organ *in vitro* systems with biological relevant flow rates that recapitulate the physiology and compartmentalization in the microenvironment of organs. These microfluidic devices have been applied to therapeutic areas ranging from chronic kidney disease (CKD) to central nervous system disorders (Brown et al., 2020). In this review, hPSC kidney organoids and MPS platforms will be discussed.

### Kidney Organoid Systems

Organoids are commonly generated using induced differentiation of human pluripotent stem cells. Increased understanding of organogenesis of multiple human tissues have led to the development of several organoid systems; potentially, there are as many organoid systems as there are tissue systems. Over the last decade, intestinal, cerebral cortex, pituitary, lung, and kidney organoids, among others, have been developed, providing complex developmental models of these tissues (Clevers, 2016). As importantly, organoids also allow insight into different disease states as well as how toxins and drugs might affect organ function.

In the last decade, organoids have become a critical tool for studying various diseases that affect the kidneys as well as a pre-clinical model for drug screening and development. Through systematic exposure to growth factors and signaling molecules that mimic embryonic environments, researchers can derive kidney organoids from human pluripotent stem cells (hPSC). Due to the numerous cell types present in kidneys, the various protocols that have been developed result in the production of kidney organoids with varying complexity and number of cell types present. (Freedman et al., 2015; Morizane et al., 2015; Takasato et al., 2015; Ciampi et al., 2016; Sharmin et al., 2016; Przepiorski et al., 2018; Kumar et al., 2019; Low et al., 2019; Tsujimoto et al., 2020). Diseases affecting specific cell types can be better replicated and studied in a more complex setting that considers important environmental factors such as cell-cell interactions and extracellular matrix effects than traditional 2D models.

One predominant kidney condition that has been modeled and studied using organoids is autosomal dominant polycystic kidney disease (ADPKD). ADPKD is a common renal genetic condition that causes cyst formation and expansion in the kidneys and can result in kidney failure and necessitate renal replacement therapy via transplant or dialysis. The disease is characterized by mutations in the PKD1 and PKD2 genes (The European Polycystic Kidney Disease Consortium, 1994; Mochizuki et al., 1996). Kidney organoids have allowed investigators to probe ADPKD by creating human knockout models that mimic cyst formation observed in patients. Freedman et al., for example, used CRISPR-Cas9 gene editing to produce PKD1 and PKD2 knockout models (Freedman et al., 2015; Cruz et al., 2017; Shimizu et al., 2020). These models accurately recapitulate the cystogenic phenotype observed in patients with ADPKD. More importantly, these models have allowed for further investigation of the roles that other factors may have on disease etiology; the role that the extracellular environment plays on cyst formation, for example (Cruz et al., 2017). In addition to ADPKD, numerous other nephropathologies are being investigated using kidney organoids such as mucin1 kidney disease and podocytopathies (Kim et al., 2017; Hale et al., 2018; Dvela-Levitt et al., 2019). There are many kidney diseases, most of which lack treatment options, and there is great potential in using organoids to better model these diseases and gain valuable insight that would lead to better understanding of the disease and novel treatment targets.

Kidney organoids also have the potential to be useful tools in pre-clinical investigation of the effects, efficacy, and safety of potential therapeutics. To date, there are few publications on current usage of kidney organoids as preclinical models for drug development, however, they may provide advantages over traditional 2D cell culture. One benefit is the presence of multiple cell types. 2D cell cultures are typically limited to one or two cell types cultured in a flask or dish. Investigational drugs have some potential of being nephrotoxic to more than one cell type; for an organ with numerous cell types, all of which potentially interact with each other, organoid systems offer better insight into the benefits and detriments that potential therapeutics may have on the kidney. This is important when considering that the kidney is also a site of drug metabolism and excretion; regardless of whether the kidney is the therapeutic target, new drugs are highly likely to pass through and possibly accumulate in the kidney resulting in organ toxicity. Cultures containing a single cell type may miss critical toxicological information if critical bioactivating or clearance enzymes are absent in that cell. Another key advantage of organoids is that cells can be cultured for longer periods of time than 2D cultures while retaining cell type specific phenotypes, e.g., tubular epithelial, glomerular, etc. Immortalized kidney cell lines, which can be cultured indefinitely, typically do not display key phenotypes of any specific cell type (Van der Hauwaert et al., 2014). Primary 2D cell lines, on the other hand, lose key expression characteristics over time and are therefore inadequate for long term *in vitro* studies (Zhang et al., 1991). Lastly, protocols have been developed that allow for bulk and automated hPSC induction and organoid culture, which could allow for high throughput screening using a relevant kidney model, something that is currently not available for 2D cell cultures (Astashkina et al., 2012; Czerniecki et al., 2018; Przepiorski et al., 2018; Kumar et al., 2019). Despite no preclinical evidence as a drug development tool, several groups have demonstrated that kidney organoids have the potential for drug development and toxicity screening. hPSC-derived kidney organoids recapitulate the toxicity observed from treatments with known nephrotoxic agents such as doxorubicin, aminoglycosides, and cisplatin (Hale et al., 2018; Kumar et al., 2019; Digby et al., 2020; Lawlor et al., 2021). Further validation and testing need to be performed however, and when combined, these advantages over traditional 2D cell culture make organoids a powerful tool for drug discovery and toxicity screening.

In addition, kidney organoids also have potential applications in regenerative therapy. Replacing cells lost in kidney diseases by using organoids derived from the patient’s own PSCs remains an attractive prospect, particularly in end stage kidney disease. Currently, dialysis and transplantation are the primary treatments but are accompanied by complications, immune system issues, organ donor shortages and high mortality rates (Abbasi et al., 2010). Theoretically, as organoids would be derived from the patient themselves, immunogenic responses would not be an issue and organoids would bypass limitations in donor organ availability. While there have been no definitive studies regarding the successful use of kidney organoids as effective treatments in patients with end stage kidney disease, several groups have reported positive results inducing PSCs into kidney organoids and transplanting them onto animal models (Sharmin et al., 2016; Van den Berg et al., 2018). These preliminary data are encouraging for potential application of kidney organoids as therapeutic interventions; however, caution should be taken moving forward. Recent work done by Nam et al. raised several safety concerns, including issues in maturation of grafted organoids as well as contamination of non-renal cell types (Nam et al., 2019). These concerns need to be addressed and overcome before attempts in humans are made. A safe and efficacious process for transplanting organoids into humans needs to be developed and potential short- and long-term adverse effects of such a process would need to be thoroughly evaluated. Nevertheless, organoids remain a viable novel therapeutic for the treatment of end stage kidney diseases.

Many challenges and hurdles remain prior to the widespread use of organoid technology. The development and validation of organoid models for disease states and as screening tools for efficacy and safety is moving quickly but will take time. Finally, use of kidney organoids in regenerative therapy has yet to be demonstrated and properly assessed for short- and long-term success and safety. Further studies are needed to determine the full extent of kidney organoid applications in these areas. Despite these limitations, kidney organoids have great potential and provide new and exciting opportunities in biomedical research and clinical medicine.

### Kidney Microphysiological Systems

MPS are able to replicate multiple tissue and organ functions in the human body, including the kidney proximal tubule to evaluate tubular secretion and nephrotoxicity, the blood brain barrier to mimic neurovascular function, and the intestinal epithelium to monitor the kinetics of absorption, transport, and drug metabolism. (Yeung and Himmelfarb, 2019). Kidney MPS systems replicate kidney function better than traditional 2D cell culture due to the presence of flow-mediated fluid shear stress and mechanical strain which replicate urinary flow and drive proper cellular morphology (Maass et al., 2017; Lee and Kim, 2018). Shear stress is also critical for proper trafficking and expression of apical and basolateral transporters-proteins critical for drug disposition and drug-induced toxicity in the proximal tubule (Fukuda et al., 2017). A glomerulus MPS is also in development, despite the high degree of structural tortuosity that is challenging to replicate. Petrosyan et al. have created a glomerulus MPS that reproduces the functional glomerular filtration barrier *in vitro* by co-culturing podocytes and glomerular endothelial cells. (Petrosyan et al., 2019). To date, most reported kidney MPS models are limited to PTECs to model drug disposition and nephrotoxicity (Sakolish et al., 2018).

MPS systems consisting of kidney tubular epithelial cells are useful for the evaluation of the disposition and toxicity of drugs or environmental chemicals. Microfluidic systems have been used to evaluate kidney-specific injury biomarkers and conduct transport and metabolic function studies (Lee and Kim, 2018; Phillips et al., 2020). Kidney MPS have also been used to screen for efficacy of investigational therapeutic agents. Weber et al. (2018) reported the use of a human kidney MPS to conduct safety testing of new chemical entities while defining the toxicological pathway. Using a PTEC MPS, they observed increases in kidney injury molecule-1 (KIM-1), urinary protein, and miRNA biomarkers caused by of polymyxin B exposure (Weber et al., 2018). Chapron et al. applied the same human PTEC MPS to elucidate the importance of renal megalin in vitamin D homeostasis (Chapron et al., 2018).

While most MPS platforms contain a single cell type, advances in bioengineering have facilitated the co-culture of multiple cell types. Lin et al. reported a 3D vascularized proximal tubule MPS (VP-MPS) that demonstrated active reabsorption of albumin and glucose via crosstalk between tubular epithelial and vascular endothelial homeostasis in kidney disease (Lin et al., 2019). Similarly, Chapron et al. also reported the construction of a dual-channel MPS co-cultured with human PTECs and human umbilical vein endothelial cells to model a vascularized kidney proximal tubule with renal vessel and tubule structures. A common feature shared by many reported dual channel VP-MPS is an “interstitial” matrix that enables solute travel between a vascular endothelial channel and an adjacent tubular epithelial channel via extravasation and diffusion. The co-culture system described by Chapron et al. exhibited cell marker expression of endothelial CD-31 and tight junction protein marker ZO-1 confirming intact cellular barrier formation, as well as expression of Na+/K + ATPase and organic anion transporter (OAT) 1 localized to the basolateral aspect of the cell. This work also confirmed active tubular secretion of a prototypical anionic drug substrate, p-aminohippuric acid (Chapron et al., 2020).

Physiologically based pharmacokinetic (PBPK) modeling is a quantitative computational method that simulates the processes of absorption, distribution, metabolism, and excretion with a set of mathematical equations to predict *in vivo* drug plasma or urine concentrations over time. Successful PBPK modeling requires accurate *in vitro* estimates of pharmacokinetic parameters for compounds of interest (Abaci and Shuler, 2015). Experimental data derived from MPS, in combination with mathematical *in silico* modeling has enormous potential to accelerate drug development. Sakolish et al. combined PTEC MPS data with PBPK models for polymyxin B, cisplatin, and gentamicin to reproduce renal reabsorption kinetics and predict renal clearance *in vivo* (Sakolish et al., 2020). In a complimentary study, Maass et al. paired a kidney MPS with a computational quantitative systems pharmacology (QSP) model to assess drug-induced renal toxicity. The prediction of clinical plasma and urine levels of KIM-1 were based measurements of *in vitro* KIM-1 with relevant drug concentrations of known nephrotoxins cisplatin, rifampicin, and gentamicin. Using QSP modeling coupled with MPS experimental data, the investigators were able to optimize clinical dosing regimens of novel compounds and predict toxic drug concentrations (Maass et al., 2019).

In addition to the prediction of clinical dosing parameters, kidney MPS can be used to study pathways of nephrotoxicity. Recently, Imaoka et al. used a PTEC MPS to investigate the mechanism of Ochratoxin A (OTA)-induced kidney injury. In this study, PTEC MPS were perfused with clinically observed concentrations of OTA. A significant decrease in OTA-induced toxicity was observed with administration of ABT (1-aminobenzotriazole), a pan-inhibitor of P450; OTA-induced toxicity was enhanced by treatment of NBDHEX (6-(7-Nitro-2,1,3-benzoxadiazol-4-ylthio) hexanol), an inhibitor of glutathione S-transferase enzymes. Taken together, these studies confirmed the ability of the PTEC MPS system to detoxify or bioactivate OTA. In addition, VP-MPS was used to confirm that basolateral OTA uptake was mediated primarily by OAT 1/3 transporters. (Imaoka et al., 2020). Yin et al. developed a platform comprised of three polydimethylsiloxane (PDMS) layers interleaved PTECs and peritubular capillary endothelial cells; this system was able to effectively produce a concentration gradient and monitor membrane permeability and has the potential to serve as model for drug nephrotoxicity evaluation (Yin et al., 2020).

Many other uses of MPS platforms have been described. For example, determining the effect of human serum albumin (HSA) on OAT1 mediated active transport of the highly albumin-bound uremic solute, indoxyl sulfate (IxS). For this study, a kidney MPS was used to evaluate IxS renal uptake by OAT1 in the presence and absence of HSA or CKD-modified HSA. IxS uptake kinetic parameters, including OAT1 affinity and intrinsic renal clearance, were incorporated with *in vitro*–*in vivo* extrapolation to predict IxS renal clearance at different stages of CKD. (Van der Made et al., 2019). In a second novel use of MPS technology, Wang et al. used a three-layered distal tubule microfluidic chip to examine pseudorabies virus-induced renal dysfunction in the kidney; this work suggested that kidney MPS may also play a role in the investigation of viral pathogenesis (Wang et al., 2019). Furthermore, Schmieder et al. (2019) presented a MPS with an artificial glomerular filtration barrier that demonstrated that increased protein transport resulted in cellular barrier permeation and barrier function damage. The interaction between high blood pressure and the integrity of the glomerular filtration barrier was evaluated; the authors concluded that increased pressure caused by substrate transport was implicated in cellular damage and increasing barrier permeability.

Taken together, the development of kidney MPS allows researchers to elaborate critical scientific questions and overcome the drawbacks of static 2D cell cultures and animal models. The physiologically relevant properties of MPS support further research in the contexts of drug efficacy, drug and drug interactions, and drug-induced nephrotoxicity. Potential future directions may include evaluation of the regenerative properties of the renal tubular epithelium. (Sura et al., 2020). Moreover, connected multi-organ MPSs could greatly improve simulation of clinical outcomes and pharmacokinetics by coupling with *in silico* pharmacokinetic modeling and provide an alternative approach to guide first-in-human clinical trials.

## Discussion

**FIGURE 1:**
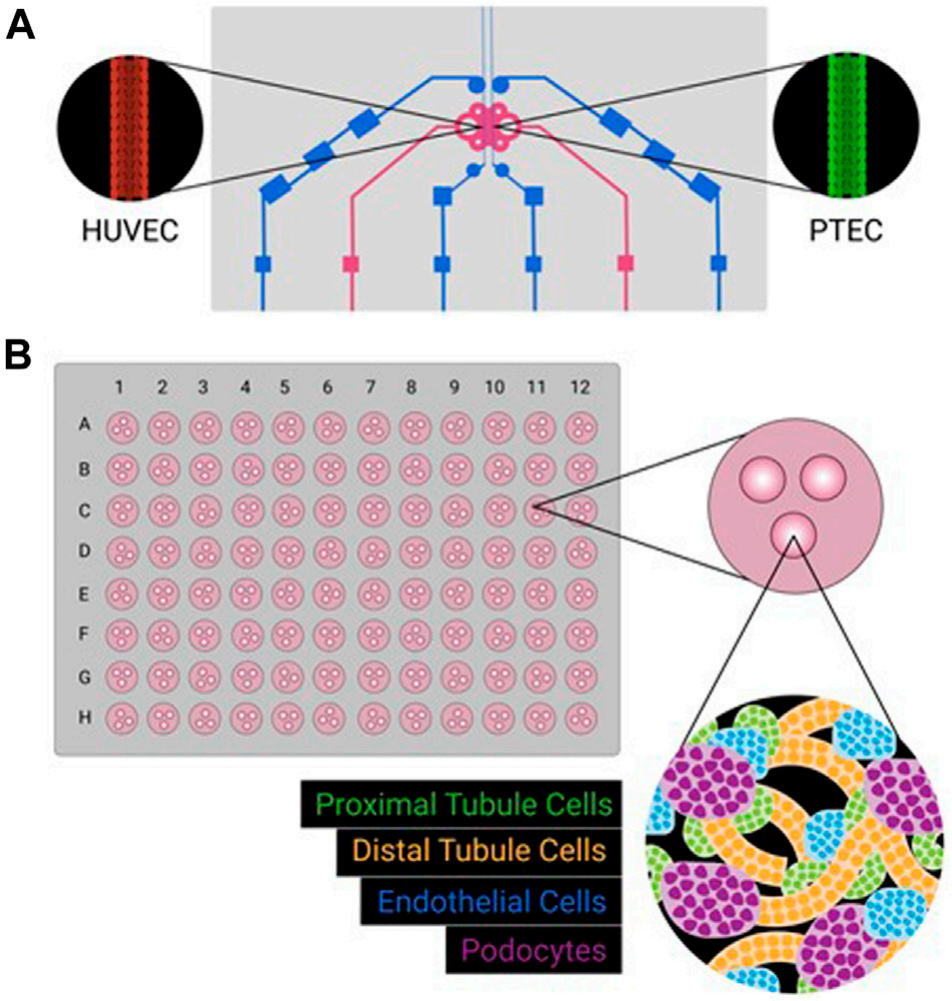
Schematic representations of kidney MPS **(A)** and organoid system **(B)**. Kidney MPS often culture a single cell type, sometimes adjacent to a second cell type, on a three-dimensional platform mimicking physiological conditions (e.g., tubular structure, constant flow, shear stress). Kidney organoids can be cultured in large batches (e.g,. a 96-well format), with multiple cell types emerging from iPSC sources. While different cell types will self-organize, formation complex physiologically relevant three-dimensional structures is incomplete.

In this short review, we have presented two models of 3D kidney cell cultures: organoids and microfluidic chip systems (Figure 1). Both systems, having their own unique benefits and disadvantages (Table 1), have exciting applications in the field of drug development and discovery. Organoids are advantageous due to their multi cell-type nature, longevity in culture, and the potential for automation resulting in high throughput applications. However, the kidney is a highly perfused organ and the lack of a functional vasculature system in the kidney organoids present a limitation for the study of the interactions with the vascular endothelium and the replication of kidney diseases of vascular origin. In addition, it is unclear if proper immune response can be elicited in kidney organoids. Significant batch-to-batch variability can also be problematic, resulting in inconsistent size and content of nephron-like structures. This is in contrast with kidney MPS, specifically PTEC MPS, which have defined 3D tube structures that properly mimic *in vivo* proximal tubule structures. Kidney MPS are also less variable from batch-to-batch that are typically cultured with a small number of cells. This simplicity is both an advantage and disadvantage-individual enzymes or transporter proteins can be studied, but in a contrived non-biomimetic environment that does not include physiological multi-cell interactions. Like organoids, PTEC MPS are viable for more than 28 days while maintaining a functional epithelial phenotype; moreover, MPS co-cultured with podocytes and glomerular endothelial cells also show extended cell viability compared with traditional 2D cell cultures (Weber et al., 2016; Petrosyan et al., 2019). More work needs to be done but the utility of these 3D cell culture technologies has an optimistic outlook and the potential to accelerate drug development while reducing the use of animal testing.

**TABLE 1:** Kidney 3D culture models.

	Organoids	Microphysiological systems (MPS)
Advantages	• Derived from human pluripotent stem cells (hPSCs) allowing models of disease states and genetic variability	• Engineered microchips with primary human kidney cells to mimic kidney functions
	• Contains more than 1 cell type allowing simultaneous toxicity screenings of multiple cell types	• Recapitulate fluid shear stress and mechanical strain
	• Recapitulates interactions between different cell types present in the system	• Media to cell ratios approximate physiological values
	• Cells maintain proper gene expression and phenotypes longer than traditional 2D cultures, allowing for longer treatments and studies	• Under consideration by pharmaceutical industry and regulatory agencies
	• Ability to respond to stress by expressing and/or releasing injury markers in specific cell types	
	• Can be automated allowing for higher-throughput screening	
Disadvantages	• Lack of vascularization	• Inconsistent reproducibility due to variability from both donors and suppliers
	• Lacks physiologically relevant components such as fluid flow	• Lack of standardized kidney chip format allows more variability
	• Limited ability to grow and mature	• The majority of reported kidney MPS models are limited to PTECs
	• Differences in hPSC sources and differentiation protocols could lead to batch-to-batch variability, affecting experimental reproducibility	• High cost of a single MPS platform
		• Potential adsorption of test agents by PDMS in many systems
		• *In vitro-in vivo* translation requires optimization in multi MPS scaling
Culture time	• Slower (weeks to months) to establish due to lengthy hPSC induction	• Faster (days to weeks)
